# Studying ancient human oral microbiomes could yield insights into the evolutionary history of noncommunicable diseases

**DOI:** 10.12688/f1000research.129036.1

**Published:** 2023-01-30

**Authors:** Abigail S Gancz, Laura S Weyrich

**Affiliations:** 1 Department of Anthropology, Pennsylvania State University, State College, PA, 16802, USA; 2 Huck Institutes of the Life Sciences, Pennsylvania State University, State College, PA, 16802, USA; 3 School of Biological Sciences, University of Adelaide, Adelaide, South Australia, 5005, Australia

**Keywords:** ancient health, oral microbiomes, dental calculus, NCDs, frailty

## Abstract

Noncommunicable diseases (NCDs) have played a critical role in shaping human evolution and societies. Despite the exceptional impact of NCDs economically and socially, little is known about the prevalence or impact of these diseases in the past as most do not leave distinguishing features on the human skeleton and are not directly associated with unique pathogens. The inability to identify NCDs in antiquity precludes researchers from investigating how changes in diet, lifestyle, and environments modulate NCD risks in specific populations and from linking evolutionary processes to modern health patterns and disparities. In this review, we highlight how recent advances in ancient DNA (aDNA) sequencing and analytical methodologies may now make it possible to reconstruct NCD-related oral microbiome traits in past populations, thereby providing the first proxies for ancient NCD risk. First, we review the direct and indirect associations between modern oral microbiomes and NCDs, specifically cardiovascular disease, diabetes mellitus, rheumatoid arthritis, and Alzheimer's disease. We then discuss how oral microbiome features associated with NCDs in modern populations may be used to identify previously unstudied sources of morbidity and mortality differences in ancient groups. Finally, we conclude with an outline of the challenges and limitations of employing this approach, as well as how they might be circumvented. While significant experimental work is needed to verify that ancient oral microbiome markers are indeed associated with quantifiable health and survivorship outcomes, this new approach is a promising path forward for evolutionary health research.

## Introduction

Noncommunicable diseases (NCDs) (e.g., chronic conditions that are not the result of a singular infectious agent) are among the leading causes of death worldwide (WHO 2020). Specifically, seven of the top ten causes of death in 2019 were attributed to NCDs, and these accounted for 44% of all global deaths. Yet, despite the devastating emotional and economic impacts of these conditions, much remains unknown about the history of these diseases or how past dietary, lifestyle, and environmental factors modulated their prevalence and impacts in different populations. To gain a fuller understanding of why these conditions vary across individuals and populations today, it is imperative to understand the patterning of these conditions across space and time. Such research would benefit our understanding of disease origins, etiologies, and prevention strategies, especially for non-Western, traditionally marginalized groups.

While infectious diseases with specific biological pathogens such as tuberculosis ( Donoghue 2017) or bubonic plagues ( Bos *et al*. 2011; Spyrou *et al*. 2019) have benefitted from recent improvements in the abilities of researchers to recover, authenticate, and analyze ancient DNA (aDNA), research into NCDs and their health impacts has lagged behind. This trend has emerged for multiple reasons. First, the vast majority of NCDs leave no distinctive skeletal traces, while individuals with infectious diseases can often be identified through distinctive mass-death burials or specific skeletal pathologies. Moreover, unlike with infectious diseases, aDNA cannot be directly used to establish the clear presence of a single pathogenic agent. As such, identifying the presence of NCDs in a population, let alone diagnosing NCDs in specific ancient individuals, is extremely difficult in comparison.

This challenge is one that may now begin to be addressed by examining the human oral microbiome. The oral microbiome consists of the microscopic organisms (e.g., bacteria, viruses, fungi, archaea, protozoa) that colonize the teeth, gums and other tissues of the mouth ( Gomez and Nelson 2017). While a core microbiome exists across most individuals, significant variation can arise depending on an individual’s unique environment, lifestyle, and physiology ( Deo and Deshmukh 2019; Verma, Garg, and Dubey 2018; Gomez and Nelson 2017; Weyrich 2021). A natural part of the human body, the oral microbiome performs several critical functions underlying systemic health including pathogen inhibition, immune system training and regulation, nutritional absorption, and enhancement of metabolic uptake ( Wade 2013). In addition, through both direct and indirect pathways, the oral microbiome modulates the risks and severities of local and systemic human diseases. Indeed, over the last decade, it has become increasingly evident that the oral microbiome has important immunological and mechanistic functions associated with NCD risk. Specifically, the relationships between the oral microbiome and cardiovascular conditions, diabetes mellitus, rheumatoid arthritis, and Alzheimer's disease have been well-established. By curating the oral microbiome features associated with the presence of these NCDs and testing their association with quantifiable indicators of health and survivorship in ancient populations, researchers can now begin to explore the possibility of identifying facets of hidden, NCD-associated morbidity and mortality risk within these groups.

Differential health risks and trends associated with ancient oral microbiomes have already begun to emerge. Specifically, an analysis of over 127 Medieval and Post-medieval individuals from the city of London conducted by (Gancz *et al*. in-review) found clear associations between systemic health associated skeletal traits including non-specific periostitis, joint porosity, and osteophytic lipping and oral microbiome community features. These findings highlight that specific markers of the oral microbiome are indeed associated with disease risk. In the following review and theoretical perspective, we highlight future steps that should be taken to improve upon and utilize these associations for the benefit of ancient health research.

## Background

The human microbiome encompasses several distinct communities of microbes that exist on nearly every surface of the body, most abundantly in the gut and mouth ( Ursell *et al*. 2012). In recent years, researchers have explored the associations of these microbiomes with human migration, evolution, culture, and, importantly, health (as reviewed in Vangay *et al*. 2018; Sharma *et al*. 2018; Weyrich 2021). The relationship between health and the microbiome has been explored using animal models, examinations of close evolutionary relatives (i.e., comparisons across primates), and research on how native or indigenous lifestyles influence the microbiome differently from those in Western societies (e.g., Liddicoat *et al*. 2020; Dent, Berger, and Griffin 2020; Janiak *et al*. 2021).

Although the gut microbiome is the most extensively studied microbiome, the oral microbiome has also begun to elicit considerable interest from researchers and the public for its connections with a number of systemic conditions. The oral microbiome comprises millions of microbes, including over 700 species of bacteria, known to colonize both the soft and hard tissues of the oral cavity ( Kilian *et al*. 2016). The establishment of these microbes begins directly after a child’s birth and continues through early childhood. Initial colonization begins with pioneer species, and once tooth eruption begins, a more complex microbial community is established on the hard surfaces ( Deo and Deshmukh 2019). While a core microbiome consisting of *Streptococcus*, *Lactobacillus*, *Actinomyces*, *Neisseria,* and *Veillonella* bacteria is common across individuals, significant variation can arise depending on an individual’s unique environment, lifestyle, physiology, and heritage ( Deo and Deshmukh 2019; Verma, Garg, and Dubey 2018; Gomez *et al*. 2017; Weyrich 2021; Handsley-Davis *et al*. 2022). The oral microbiome performs several critical functions underlying systemic health. Specifically, human oral microbes are involved in pathogen inhibition, immune system training and regulation, nutritional absorption, and the enhancement of metabolic uptake ( Wade 2013; Shaw, Smith, and Roberts 2017). These functions have evolved alongside humans over time with shifting environments, diets, and behaviors ( Weyrich 2021).

Unlike other microbiomes, the oral microbiome can also be reliably reconstructed in ancient populations from calcified dental plaque, also known as dental calculus. Calculus forms during life and shares similarities with a living individual's oral microbiome ( Velsko *et al*. 2019). This biological substance accumulates over the lifespan as oral microbiota organize into complex biofilms macroscopically observable as plaque ( Welch *et al*. 2016). Over time, salivary calcium phosphate salts cause the biofilm to calcify, thereby encapsulating microorganisms, food debris, proteins, and other materials within ( Weyrich, Dobney, and Cooper 2015). Through ancient metagenomic ( Warinner, Speller, and Collins 2015; Weyrich, Dobney, and Cooper 2015), proteomic ( Jersie-Christensen *et al*. 2018; Hendy *et al*. 2018), isotopic ( Eerkens *et al*. 2014), and other forms of analysis, dental calculus has been used to study human migrations ( Eisenhofer and Weyrich 2018), subsistence practices ( Adler *et al.* 2013) and disease ( Yaussy and DeWitte 2019).

The oral microbiome is a major driver of both oral and systemic health. In the mouth, the oral microbiome is associated with two of the most common dental health conditions, specifically caries (cavities) and periodontitis. Caries are associated with tooth decay, which is caused by the breakdown of enamel, and are a major public health problem today (Heng 2016). Their etiology is associated with dietary, environmental, behavioral, developmental, and genetic factors. Several bacterial genera, including *Streptococcus*, *Lactobacillus*, *Actinomyces*, *Fusobacterium*, *Porphyromonas*, *Selenomonas*, *Bifidobacterium, Veionella* and *Scarvoia* have been associated with caries ( Simón-Soro and Mira 2015; Tanner *et al*. 2011; Skelly *et al*. 2020; Handsley-Davis *et al*. 2020). The state of disease is not solely linked to the composition of the microbiome but also to its functional activities ( Solbiati and Frias-Lopez 2018) and the ways that microbes interact with each other (e.g., suppressing colonization by competitors) ( Sharma *et al*. 2018). Periodontitis is a form of gum infection that is associated with both soft and hard tissue destruction, as well as eventual tooth loss. Some common examples of bacterial species in the periodontal microbiome include *Porphyromonas gingivalis*, *Tannerella forsythia, Treponema denticola*, *Prevotella intermedia*, *Eikenella corrodens*, *Fusobacterium nucleatum*, and *Aggregatibacter actinomycetemcomitans* ( Pritchard *et al*. 2017). Like caries, there are also key functional traits (e.g., upregulation of virulence factors) associated with periodontitis ( Solbiati and Frias-Lopez 2018).

While these local health outcomes of the oral microbiome are of significant research interest in and of themselves, these oral diseases have been shown to be strongly correlated with, and at times specific risk factors for, systemic disease ( Handsley-Davis *et al*. 2020). For example, caries have been used as an indicator of systemic diseases ( Dashper *et al*. 2019), and periodontitis has been linked to a multitude of other conditions and systematic effects likely mediated by inflammation ( Hajishengallis 2015; Genco and Borgnakke 2020). In fact, systemic inflammation and periodontitis are known to form a positive feedback loop by which one exacerbates the other ( Akcali *et al*. 2013). Despite these associations, there are also direct mechanisms by which the oral microbiome can contribute to systemic diseases independently of oral diseases. Three main mechanisms have been proposed for how the oral microbiome is able to impact the rest of the body: (1) the translocation of oral microbes into other regions, (2) the translocation of oral microbiome metabolites, and (3) the instigation of immunological and inflammatory modulations that have systemic effects ( Hajishengallis 2015; Thomas *et al*. 2021; Kleinstein, Nelson, and Freire 2020; Park *et al*. 2022; Bowland and Weyrich 2022). Via these mechanisms, various NCDs have been shown to be directly caused by the oral microbiome; these interactions are described in greater detail in the following sections.

The observation that oral health and systemic health outcomes are related has already been utilized for research into ancient human health. Specifically, macroscopically observable differences in oral health and calculus formation have been studied by archeologists as a marker of frailty (i.e., heightened susceptibility to different diseases and stressors and their risks of death) ( DeWitte and Stojanowski 2015). Dental calculus and other indicators of oral health have been effectively used as overall indicators of general health and disease risk in past populations ( DeWitte and Bekvalac 2010; Yaussy and DeWitte 2019; Hakeem, Bernabé, and Sabbah 2019), although these data have not yet been directly linked to specific microbial or immunological mechanisms. By analyzing the microbiome of individuals, it may be possible to further identify the specific markers of frailty within ancient populations and define some of the microbially-modulated mechanisms by which health outcomes occur. This technique would offer novel approaches to addressing the osteological paradox, a major challenge of paleoepidemiological research, specifically the issue of heterogeneity in frailty, as described by DeWitte and Stojanowski (2015).

**Figure 1.**
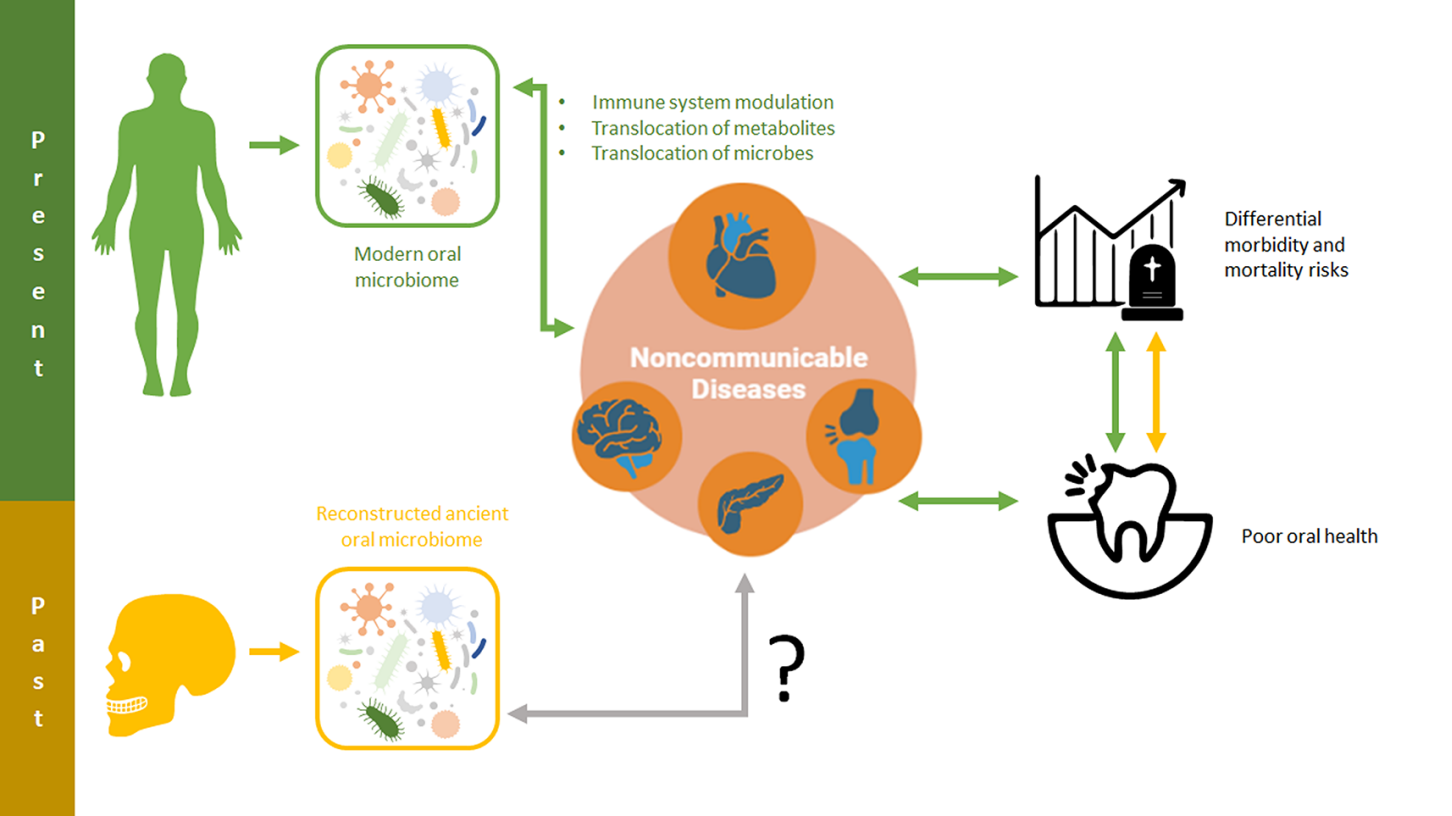
The human oral microbiome can lead to systemic effects, including the onset and modulation of noncommunicable diseases, via immune system modulation, the translocation of microbe metabolites, and the translocation of microbes. These relationships and the consequences they have for oral and overall health may be critical for shedding light on noncommunicable disease risk and impacts in past populations.

In the following sections, we review some of the most common NCD in modern populations and curate oral microbiome markers that could be applicable to ancient populations ( Figure 1).

## Systemic disease and the oral microbiome

### Cardiovascular disease

Cardiovascular disease (CVD) is a broad category of conditions that includes congestive heart failure, cardiac arrhythmias, valvular heart disease, stroke, coronary heart disease, atherosclerosis, myocardial infarctions, and strokes. Today, CVD is a leading cause of death both worldwide and in the United States ( Lin *et al*. 2020). In the US alone, CVD accounts for almost one in every four deaths ( Benjamin *et al*. 2017). While many risk factors exist for CVD, such as obesity, hypercholesterolemia, sex, age, hypertension, smoking, and genetic predispositions, many patients do not exhibit these predispositions ( Frias-Lopez and Duran-Pinedo 2020). Multiple studies have demonstrated the linkages between oral health and CVD risk ( Buhlin *et al*. 2002; Meurman, Sanz, and Janket 2004; Jansson *et al*. 2001; Mattila *et al*. 1989; Dietrich *et al*. 2017). For example, the prevalence and incidence of coronary heart disease is significantly increased in periodontitis according to a meta-analysis of five cohort studies and 86,092 patients ( Bahekar *et al*. 2007). Moreover, periodontal interventions have been shown to reduce the risk of CVD ( Roca-Millan *et al*. 2018).

While the links between oral health and CVD are more established than that of CVD and the oral microbiome, several studies have still demonstrated clear associations between these factors. For example, in 2011, Figuero and colleagues scraped atheromatous (arterial) plaque from the carotid arteries of patients and used 16S rRNA sequencing methods to evaluate bacterial DNA ( Figuero *et al*. 2011). The researchers found periodontitis-associated bacteria within the plaque, including *P. gingivalis*, *A. actinomyctemocitans*, *T. forsythia*, *E. corrodens*, *F. nucleatum*, and *Campylobacter rectus.* In 2011, Koren and colleagues used the same approach to look at bacteria in athersclerotic plaque, oral, and gut samples in a case control study of 15 patients ( Koren *et al*. 2011). They found that the abundances of *Villanella* and *Streptococcus* species were correlated between the oral cavity and atherosclerotic plaques. Both of these studies are important because they demonstrate a possible mechanism linking oral microbes to CVD, wherein oral microbes invade the bloodstream and result in inflammation, atherosclerosis, and other risk implications. Animal models also support the linkage between CVD and oral microbes. In 2014, Velsko and colleagues published on hyperlipidemic mice that were infected with *P. gingivalis* and *T. denticola* ( Velsko *et al*. 2014). The researchers demonstrated that infection was associated with alveolar bone loss, aortic atherosclerosis, and an induced immune response ( Velsko *et al*. 2014). Within all of these studies, the differences observed in *Streptococcus* are especially remarkable, as specific *Streptococcus* species are thought to be directly associated with CVDs ( Shi *et al*. 2021; Chen *et al*. n.d.; Nomura *et al*. 2020). Specifically, certain species of *Streptococcus* have been directly observed to bind to host cardiovascular tissues and contribute to diseases such as endocarditis. In addition, other species of *Streptococcus*, specifically Group A (GAS), can lead to rheumatic fever, which also contributes to CVDs ( Coffey, Ralph, and Krause 2018).

As a whole, this research indicates that there are strong associations between the oral microbiome and CVDs. These relationships are facilitated by two major mechanisms. The first of these is bacterial escape from the oral cavity, and the second is via inflammatory pathways. Specifically, oral microbes associated with periodontitis can reach the vascular system, invade local cells, and be associated with CVD ( Reyes *et al*. 2013). Via inflammatory pathways, microbes inhabiting dental plaque can stimulate cytokine production and elevate their levels in the blood, leading to inflammatory responses ( Dietrich *et al*. 2017). These mechanisms suggest that there are both specific and nonspecific oral microbiome traits associated with CVDs. Nonspecifically, oral microbiome taxa and functions associated with increased periodontitis or systemic inflammation may serve as a proxy for CVD risk. Specifically, the presence or a specific abundance of *Streptococcus* species capable of binding to cardiovascular tissues may serve as a direct risk factor. Indeed, it may even be possible for ancient health researchers to sample dental pulp chambers as a way to investigate whether CVD-associated taxa were present in the circulatory system of an individual. As such, these oral microbiome features suggest that it may well be possible to curate a proxy for CVD-risk using microbial traits.

### Diabetes

Diabetes mellitus (DM) describes a set of metabolic disorders associated with blood sugar dysregulation. Approximately 451 million adults (5.9% of the global population) in 2017 were estimated to be living with DM, with the disease burden increasing annually ( Lin *et al*. 2020). Individuals living with DM contend with both reduced quality of life and increased rates of mortality ( Yang *et al*. 2019). Type one DM (T1D) and type two DM (T2D) are the most common and are caused by pancreatic β-cells insulin-production failure and acquired insulin resistance, respectively ( Menezes-Silva and Fonseca 2019). In T1D, this is caused by the self-destruction of insulin producing pancreatic cells by the immune system ( Boerner and Sarvetnick 2011). In T2D, bodily tissues such as the muscles and fat also develop insulin resistance, in addition to often co-occurring underproduction of insulin. Both T1D and T2D are believed to have genetic, environmental, and lifestyle associated risk factors.

Of NCDs, DM is amongst those most closely associated with oral health. Oral health, particularly gingivitis and periodontitis, has long been studied in relation to both T1D and T2D. While the causal nature of the relationship (i.e., whether DM causes poor oral health, or vice versa) is challenging to deduce, it is clear that the disease is closely linked to oral microbiota composition and diversity ( Graves, Ding, and Yang 2020). A number of studies indicate that gingivitis and periodontitis rates are higher in diabetic individuals ( Genco and Borgnakke 2020; Lamster *et al*. 2008; Novotna *et al*. 2015), potentially up to three or four times for periodontitis ( He *et al*. 2015). These conditions are associated with fundamental changes in the functions of oral tissues, including bone loss, attachment degradations, and increased inflammatory cytokine expression ( Graves, Ding, and Yang 2020). These DM-associated changes impact the entire host immune system, as well as local microbes. Some research even suggests that the treatment of oral conditions is associated with glycemic control and therefore reduced DM symptoms ( Bharti *et al*. 2013; Moeintaghavi *et al*. 2012).

Recent studies have identified several aspects of the oral microbiome that are associated with DM. Specifically, Shillitoe and colleagues used RTq-PCR to analyze specific microbiota in patients with T2D before and after gastric bypass surgery (a type of weight loss procedure) ( Shillitoe *et al*. 2012). They found that the T2D patients had low rates of *Bifidobacterium* in the mouth, and that these rates increased after the procedure. Using 16S ribosomal RNA (rRNA) sequencing technology, Long *et al*. examined 98 patients with T2D, 99 patients who were obese and did not have DM, and 97 normal weight patients ( Long *et al*. 2017). The researchers found that *Actinobacteria* was significantly less abundant among diabetics, and that within this phylum, five families and seven genera were significantly less abundant. Kampoo and colleagues also harnessed 16S to examine T2D patients in Southern Thailand ( Kampoo *et al*. 2014). These researchers found that in the supragingival plaque of the diabetics, total levels of *Streptococci* and *Lactobacilli* were higher. In 2020, Matsha and colleagues conducted a similar study in South Africa and found that *Fusobacteria* and *Actinobacteria* were more abundant in T2D patients and *Proteobacteria* less abundant ( Matsha *et al*. 2020). Most recently in 2021, Balmasova and colleagues used 16S rRNA sequencing to find markers associated with three groups: those with chronic periodontitis associated with T2D, those with chronic periodontitis alone, and healthy controls ( Balmasova *et al*. 2021). The T2D group was associated with a lower abundance of *Streptococcus* and *Pasturellacaea* and a higher abundance of *Leptotrichicacea.* Those with T2D differed from non-T2D periodontitis patients in terms of lower abundance of *Veillonellaceae* and higher *Neisseriaceae.* Although these studies all indicate that periodontitis and a shift in oral microbiota are associated with T2D, the specific oral microbiome markers of T2D remain unclear, likely reflecting biases in methods used and populations studied.

For T1D, significant differences have also been found in the oral microbiome. In 2006, Lalla and colleagues examined 50 T1D patients from the Columbia University Diabetes Center with age, gender, and periodontal disease matched controls and found that *Eubacterium nodatum* was elevated in diabetic patients, although mostly their subgingival infection patterns were similar ( Lalla *et al*. 2006). Meanwhile, de Grot and colleagues matched 53 T1D patients with healthy controls and investigated their oral and fecal microbiota, finding that the oral microbiota were markedly different, with a high abundance of *Streptococci* and differences in composition ( de Groot *et al*. 2017). T1D individuals had higher abundances of *Actinobacteira* and *Firmicutes*, including taxa within *Streptococcus*, *Actinomyces*, and *Rothia* genera, while *Bacteroidetes* and *Proteobacteria* were increased in the controls. In 2021, Jensen and colleagues found a link between glycemic control, T1D, and the complexity and richness of plaque microbiota. This was associated with an inflammation response on a cellular level, possibly due to glycemic control interactions with the microbiome ( Jensen *et al*. 2021). From these studies, it appears that T1D is associated both with different composition as well as some alterations in taxa. More research is needed to determine whether these differential markers are consistent across populations, especially those that underwent different evolutionary selective pressures in the past.

Several mechanisms specifically linking DM and the oral microbiome have been hypothesized in the literature. The most commonly cited mechanism is systemic inflammation, which serves as a link between periodontitis, gingivitis, the oral microbiome, and systemic immune responses related to DM ( Makiura *et al*. 2008; Aemaimanan, Amimanan, and Taweechaisupapong 2013; Hyvärinen *et al*. 2015; Thorstensson, Dahlén, and Hugoson 1995; Preshaw *et al*. 2012; Levine 2013). Specifically, researchers postulate that oral microbiome in the mouths of individuals with DM, especially those in periodontal areas, causes chronic inflammation and can even trigger insulin resistance by influencing the body’s immunity. In support of this theory, Blasco-Bacque *et al*. found that mice with periodontitis had increased insulin resistance that was mediated by an adaptive immune response against oral infection ( Blasco-Baque *et al*. 2017). Glycemic control is another proposed factor, especially related to bacteria associated with periodontal disease ( Lamster *et al*. 2008). Taylor *et al*. found support for this theory in their longitudinal study of residents at the Gila River Indian Community, where they discovered that severe periodontitis was associated with the increased risk of poor glycemic control ( Taylor *et al*. 1996).

These studies demonstrate that the microbiome of patients with DM are different than those without it. However, excluding the association with periodontitis, the observed differences vary within studies. This result is not necessarily surprising, as these studies focus on several very different human populations with distinctive evolutionary histories that impact the composition of their oral microbiomes. As such, while certain changes in the abundances of taxa may be DM-associated within a specific population, these trends may not hold true in other groups. As such, DM-associated oral microbiome traits may need to be curated from modern populations related to the specific past populations researchers seek to study in order to be useful markers of DM-associated frailty. Alternatively, research into whether specific microbial functions (as opposed to taxa) are related to DM in modern groups may offer more generally applicable DM-markers.

### Rheumatoid arthritis

Rheumatoid arthritis (RA) is a chronic autoimmune disorder that impacts the joints. RA is thought to be caused by a combination of genetic, behavioral, immunological, and environmental factors ( Aho and Heliövaara 2004) The disease is more common among women than men and impacts more than 1.3 million individuals in the United States ( Rheumatoid Arthritis: Causes, Symptoms, Diagnosis & Treatments 2021). Today, RA is amongst the most prevalent of chronic inflammatory diseases ( Smolen, Aletaha, and McInnes 2016). Individuals with RA suffer from declined physical function, increased comorbidity risks, and reduced work capacity. Although RA has relatively similar prevalence in many populations, some marginalized communities have much higher incident rates ( Silman and Pearson 2002). Additional risk factors for RA include smoking, low socioeconomic status, and genetic histories ( Smolen, Aletaha, and McInnes 2016). Similar to the previously discussed NCDs, RA has been shown to be linked to periodontal disease, and numerous studies have explored the risks of RA onset and progression in relation to the oral and gut microbiomes ( Bingham and Moni 2013). While it remains unclear whether the onset of periodontal disease is a causative or correlative factor for RA, this pattern suggests strong links to the oral microbiota.

Indeed, researchers have demonstrated that there are microbial markers of RA. A good potential discriminant of RA was found in 2018 by Lopez-Olivia and colleagues. The researchers looked at 22 RA and 19 controls subgingival plaque and used 16S rRNA sequencing. PICRUSt, a tool used to predict the functional composition of a metagenome, demonstrated that arachidonic acid and ester lipid metabolism might explain clustering patterns in communities. *C. curtum*, another organism capable of producing large amounts of citrulline, emerged as a robust discriminant of the microbiome in individuals with RA ( Lopez-Oliva *et al*. 2018). In another study in 2015, Zhang and colleagues used shotgun sequencing to look at 105 oral microbiomes in RA and control patients ( Zhang *et al*. 2015). The researchers found that *Veillonella* were elevated in dental plaques of RA patients, as were *Haemophilus*, *Aggregatibacter*, *Cardiobacterium*, *Eikenlla*, *Kingella* and *Rothia dentocariosa.* Among the anaerobes found to be different were *Lactobacillus salivarius*, *Atopobium spp.*, and *Cryptobacterium curtum* (enriched in RA), as well as *Neisseria spp.* and *Rothia aeria* (decreased in RA). As a whole, the study concluded that there were differences between RA and control microbiomes. Further, Cheng *et al*. looked at subgingival plaque in healthy and diseased sites in early RA and healthy individuals. Microbial community differences were found at phylum, genus, and species levels ( Cheng *et al*. 2021). Specifically, *Capnocytophaga*, *Cardiobacterium*, *Neisseria*, and *Streptococcus* genera were all associated with RA. Likewise, Chen and colleagues found that eight oral bacterial biomarkers differentiated RA from osteoarthritis (OA) and that the microbial composition of RA, OA and healthy subjects did differ at the phylum and genus levels ( Chen *et al*. 2018). In another study, Milkuls and colleagues profiled 260 RA and 296 osteoarthritis control patients by collecting their subgingival plaque ( Mikuls *et al*. 2018). Using 16S rRNA sequencing, the researchers found that 10 different OTUs were less abundant in RA patients, including *Peptostreptococcus*, *Porphyromonas*, *Prevotella* and *Treponema* species. However, the researchers did not find associations with previously identified RA-associated oral microbes such as *A. actinomycetemomitan*s or *P. gingivalis.* As with the previously discussed diseases, these differences could be associated with differences among the populations investigated.

The mechanisms linking oral microbiomes to RA center on antibodies and other immunological factors circulating through the body and setting off an inflammatory response ( Lopez-Oliva *et al*. 2018). The translocation of microbes from the oral cavity into the bloodstream is another likely mechanism ( Huang *et al*. 2016). Another possible mechanism is the specific production of metabolic products by microbes that lead to RA formation. For example, a paper by Konig *et al* from 2016 suggested that microbes such as *A. actinomycetemcomitans* could induce hypercitrullination in host neutrophils and thus cause RA ( Konig *et al*. 2016). Studying the microbial associations between RA and the oral microbiome in the past could shed lights on these mechanisms and the specific microbial functions associated with them.

To conclude, RA has a number of possible specific microbial biomarkers, such as the presence of specific microbial functions (e.g., citrulline production) and taxa (e.g., *Veillonella, Haemophilus*, *Aggregatibacter*, *Cardiobacterium*, *Eikenlla*, *Kingella* and *R. dentocariosa*). However, it is important to note that several of these studies demonstrate contrasting results, possibly due to their focuses on populations with different evolutionary histories and microbiome structures. Therefore, more research is needed to investigate why different groups and studies return different microbes as significant biomarkers. One possibility is that different populations have different microbial structures that lead to the disease, in which case modern references for model construction should be carefully chosen by similarity to ancient samples of interest.

### Alzheimer’s disease

Alzheimer’s disease (AD) is a serious condition that currently impacts 6.3 million Americans and leads to about 120,000 deaths a year ( 2021 Alzheimer’s Disease Facts and Figures 2021). It is among the most common causes of dementia and is the sixth leading cause of death in the United States ( Weller and Budson 2018). AD is caused by the accumulation of amyloid beta plaque deposits and neurofibrillary tangles in the brain. These processes can be exacerbated by immune dysfunction caused by systemic inflammation ( Heneka *et al*. 2015). A number of studies have linked oral health to AD ( Harding *et al*. 2017; Kamer *et al*. 2020; Chen, Wu, and Chang 2017; Liccardo *et al*. 2020). For example, Chen and colleagues used a retrospective matched cohort study in Taiwan to demonstrate that chronic periodontitis and AD were risk-correlated, with patients with periodontitis having higher rates of disease than controls ( Chen, Wu, and Chang 2017).

Multiple studies have also linked the oral microbiota and AD progression. For example, in 2012, Stein and colleagues examined immunoglobulin G antibody levels for seven oral microbes ( *A. actinomycetemcomitans*, *P. gingivalis*, *C. rectus*, *T. denticola*, *F. nucleatum*, *T. forsythia*, and *P. intermedia*) in relation to AD onset and progression ( Stein *et al*. 2012). The researchers concluded that elevated antibodies associated with periodontitis could contribute to AD. A more direct investigation was conducted by Jiao and colleagues in 2019. The researchers examined 39 patients with AD and 30 healthy controls and used 16S to compare the salivary microbiome. They found a lower richness and diversity in AD patients with a relatively higher abundance of *Moraxella*, *Leptotrichia*, and Sphaerochaeta, while *Rothia* was reduced ( Jiao *et al*. 2019). Another recent study by Wu and colleagues found that AD individuals have lower microbial diversity, increased number of *Lactobacillales*, *Streptococcaceae*, *Firmicutes/Bacteroidetes*, and a significantly decreased number of *Fusobacterium* ( Wu *et al*. 2021).

The oral microbiome can influence AD outcomes through two mechanisms: the instigation of systemic inflammation and the introduction of oral microbes and their virulence factors directly into the brain through the blood-brain barrier ( Thomas *et al*. 2021; Sureda *et al*. 2020; Singhrao and Olsen 2019; Harding *et al*. 2017). Directly, oral microbes can enter brain tissue via the blood or lymphatic system and damage the neural system (e.g., Singhrao *et al*. 2015). Both these and microbial byproducts that enter the neural system can trigger inflammatory and other antibacterial responses that can promote AD ( Weaver 2020; Narengaowa *et al*. 2021). Indirectly, oral microbes can exacerbate systemic inflammation. However, the relationship between AD and the microbiome may also be bidirectional, as patients with AD are known to have limited mobility and poorer oral hygiene ( Peng *et al*. 2022).

These findings suggest specific bacterial biomarkers, such as lower diversity and the relative abundance of certain species, could be used for risk modeling of AD within populations. However, the occurrence of AD in mostly older individuals necessitates improved age estimations for archaeological applications. Specifically, point age estimations with confidence intervals, such as those acquirable via transition analysis, would be extremely invaluable ( Milner and Boldsen 2012). While individual AD-diagnosis in ancient individuals may be beyond reach, it is conceivable that the bacterial biomarkers described above could be used to detect differences in risk amongst populations.

### Other diseases

In addition to the NCDs described above, there is mounting evidence linking the oral microbiome to several other morbidities. Specifically, various types of cancers ( Frias-Lopez and Duran-Pinedo 2020; Peng *et al*. 2022), adverse pregnancy outcomes ( Frias-Lopez and Duran-Pinedo 2020), mental health disorders ( Wingfield *et al*. 2021), and other conditions have been recently explored. While this paper has focused on CVD, DM, RA, and AD, additional NCDs could be added as morbidities of interest for ancient health research.

## Applying the present to the past

With a number of specific microbial risk factors associated with NCDs evident in modern populations, an emerging frontier of ancient metagenomic research involves utilizing this information in past populations. By developing predictive risk models from the NCD data of modern populations, researchers may be able to quantify the risks associated with either specific or nonspecific NCDs in the human past ( Figure 1). Even a constrained ability to discern differential NCD-associated risk in past populations would enable researchers to ask new questions about how changing environments, lifestyles, and behaviors impacted human health. These advances are critical to pursue in order to advance the goals of the osteological paradox and improve paleoepidemiological research ( Wood *et al*. 1992; DeWitte and Stojanowski 2015). While this research is unlikely to lead to diagnostic capabilities of NCDs in past individuals, it does have the potential to reveal previously cryptic elements of frailty that are associated with the same microbial mechanisms as NCDs. That is, by identifying microbiome markers associated with higher risks of inflammation and NCDs, researchers would be better able to differentiate between the health experiences of both populations and individuals.

To implement this proposed methodology, several steps must be pursued. First, using data from modern populations (and ideally non-industrialized modern populations descended from the ancestral population of interest), specific NCDs markers, such as the ones discussed in this paper, should be curated via a meta-analysis. These markers would not need to be specific, but rather contain additive risk information regarding the likelihood of an individual or community to express NCDs. These markers may include specific taxa (e.g., species), relative diversities (e.g., low beta diversity), community structures (e.g., co-occurring groups of taxa), or microbial functions (e.g., functions related to inflammation). Next, these markers will need to be incorporated into a statistical model of risk for a given individual or population as previously done for modern individuals ( Zheng *et al*. 2020; Aryal *et al*. 2020). This would also allow researchers to explore how microbial taxa and functional profiles translate into physiological effects. Moreover, markers beyond those explored in this paper could also be utilized to explore the associations between microbes and frailty. For example, ancient metabolomics and metaproteomics could also be integrated into risk models to test if specific markers impact health and survivorship.

In ancient populations, NCD-risk models could then be validated against skeletal markers of health including age-at-death and prevalence of nonspecific skeletal stress markers. These comparisons between the oral microbiome and skeletal markers of health and survivorship would enable researchers to validate whether the oral microbiome is indeed informative about measurable aspects of frailty. Should these relationships prove valid the ancient oral microbiome approach described in this paper would not just enable risk assessment for ancient populations, but potentially provide insights into the mechanisms via which environments, behaviors, and health are linked in the modern day.

## Challenges and limitations

While this is a promising new area of exploration, we acknowledge that there are a number of challenges and limitations. For instance, archeological metadata must be improved for samples for whom ancient dental calculus is analyzed ( Gancz, Wright, and Weyrich 2022b). Specifically, improved age estimations, demographic data, and standardized paleopathological information must be collected in order for the morbidity and mortality risks associated with systemic diseases to be assessed. Without these data, it would be difficult to control for enough health-related factors to enable differences in health associated with systemic conditions be found. To elaborate on this point, the traditional age categories assigned to ancient individuals (e.g., ‘young adult’, ‘middle adult’, ‘juvenile’) are almost certainly insufficient to detect differences in survivorship associated with systemic diseases in populations. This is especially true as many of these conditions selectively impact older individuals, whose ages in the archeological record are often underestimated ( Milner and Boldsen 2012).

A deeper and more fundamental challenge of this analysis is that all the risk factors for the conditions of interest are available only for modern populations. It is known that oral microbiota changes over time ( Deo and Deshmukh 2019; Yates *et al*. 2021), and microbiota in ancient populations may not be linked with disease in the past in the same ways they are today. Moreover, these diseases may not be highly prevalent in ancient populations, which would necessitate large sample sizes. For example, RA has a prevalence of approximately 0.41–0.54 among US adults, meaning that the likelihood of obtaining a sufficient number of ancient individuals exhibiting this disease from a single tempo cultural context is low ( Hunter *et al*. 2017). Even for more widespread conditions in industrialized societies, such as obesity and diabetes, it is difficult to estimate what percentage of the population may have exhibited these diseases in the past.

Additionally, there are a number of challenges involved in the reconstruction of ancient oral microbiomes that may limit the resolution with which the suggested approach may be implemented. First, aDNA is highly fragmented and prone to contamination ( Llamas *et al*. 2017; Salter *et al*. 2014). When aDNA methodologies are applied to ancient microbes, there are additional constraints in the ability of researchers to identify specific taxa at high resolution, often due to damage as well as limited or inaccurate references ( Velsko *et al*. 2018). However, these challenges are key issues currently being addressed in the field of ancient metagenomics, and so they are likely to be mitigated as analytical procedures and bioinformatic pipelines are improved.

## Conclusions

The oral microbiome is a promising tool for understanding the mechanistic pathways via which environmental factors translate into health impacts, both today and in the past. In this paper, we suggest a novel approach for identifying NCD-associated frailty in ancient populations. The benefits of this approach are numerous as NCDs could be studied in and of themselves in association with a variety of environmental and behavioral changes over time. Moreover, the health and environmental impact of historical shifts help researchers examine why differences in disease manifestations exist in modern populations and what might be effective ways of approaching them. While substantial model development and refinement is necessary before these methods can be added into the ancient health research methodological repertoire, we consider it a promising application of ancient metagenomics.

## Data Availability

No data are associated with this article.
