# Supplementary material for: Studying ancient human oral microbiomes could yield insights into the evolutionary history of noncommunicable diseases
Source: F1000Res. 2023 Apr 6;12:109. Originally published 2023 Jan 30. [Version 2] doi: 10.12688/f1000research.129036.2 (PMC10090864; doi:10.12688/f1000research.129036.2)
Supplement: Peer review history

# Peer review: Reviewer response for version 2

**DOI:** 10.5256/f1000research.146601.r168931

**Published:** 2023-04-11

**Authors:** Taylor van Doren

**Affiliations:** 1 Sitka Sound Science Center, Sitka, AK, USA

I thank the authors for their thoughtful responses to my queries, and congratulate them on a well-written paper. I have no other comments.

Is the review written in accessible language?

Yes

Are all factual statements correct and adequately supported by citations?

Yes

Are the conclusions drawn appropriate in the context of the current research literature?

Yes

Is the topic of the review discussed comprehensively in the context of the current literature?

Yes

Reviewer Expertise:

Infectious diseases, tuberculosis, influenza, co-morbidities, biocultural anthropology, syndemics, demographic transitions, epidemiological transitions

I confirm that I have read this submission and believe that I have an appropriate level of expertise to confirm that it is of an acceptable scientific standard.

# Peer review: Reviewer response for version 1

**DOI:** 10.5256/f1000research.141691.r162486

**Published:** 2023-02-23

**Authors:** Taylor van Doren

**Affiliations:** 1 Sitka Sound Science Center, Sitka, AK, USA

This paper reviews and synthesizes the literature linking the oral microbiome to various non-communicable diseases, and additionally provides some intriguing paths forward on how to study these connections in skeletal remains to illuminate another dimension of NCDs in past human populations. I really enjoyed this interesting, well-written, and well-researched paper, and I think the authors have significant contributions to make to the body of literature on ancient human health.

 I have listed some of my more detailed thoughts below on the paper, some small and some more significant. Some of these comments are comments on mere spelling/style issues, but I have also included a couple larger items that I hope the authors consider, if not in this revision, then as the research progresses.

- First paragraph of introduction: Consider listing at least a couple of the seven of the top ten causes of death in 2019. Also, I do not suspect that the top ten causes of death would have changed substantially from 2019 to 2023 (other than the inclusion of COVID, of course), but do you have an updated source? I know that WHO has just a little bit of a lag, but there should be updated numbers from 2019 by now.
- Second paragraph of introduction: One of the reasons listed for why the trend of studying NCDs through aDNA is that “infectious diseases can often be identified through distinctive mass-death burials or specific skeletal pathologies”. Certainly, for major epidemic/pandemic events, the mass death burials will be essential, but I do not agree that infectious diseases appearing in skeletal remains would be a major contributor to this. Overwhelmingly, acute infectious disease progression is too short to leave any real traces on skeletons. Infectious diseases like tuberculosis and leprosy are (sometimes) identifiable with some difficulty in skeletal material, but only after a very long period of infection and disease progression. Skeletal pathogolies like markers of frailty could suggest potential susceptibility to acute infectious diseases, but infectious diseases overwhelmingly do not manifest in the skeleton. Because of the previous mention of Donoghue (2017), I suspect tuberculosis and leprosy are top of mind here, but this should be more carefully stated in the text.
- End of first paragraph of background: consider capitalizing “native” and “indigenous.”
- Last full paragraph on page 4: The osteological paradox is mentioned very fleetingly, and although it is a popular concept and likely well known to your audience who studies skeletal material, it might be a good idea to define it briefly. Additionally, the citation of DeWitte & Stojanowski (2015) is good, but if you do dedicate space to the osteological paradox, it would be good practice to reference the original paper (Wood et al., 1992) as well.
- First paragraph on page 5 under “Cardiovascular disease”: in the list of conditions that make up CVD, “stroke” is listed twice.
- First paragraph, 3 ^rd^ line on page 7: “ *Actinobacteira” *should be “ *Actinobacteria*”
- First paragraph in “Applying the present to the past” page 9: I see here that you do go ahead and cite the Wood et al. (1992) paper in reference to the osteological paradox! Just a small semantics thing in this sentence: I am not sure that the osteological paradox as a concept has “goals” *per se*, it would be more like the goals of understanding the nuances of the osteological paradox and the reality of the lived experiences of past populations.
- Second paragraph in “Applying the present to the past” on page 9: It occurred to me while reading this paragraph (which I really like, because it provides some clear steps forward), that the issue of the prevalence of NCDs in historical and/or prehistoric populations will be really dependent on how industrialized/urbanized/modernized a particular population is. I would not recommend going into a detailed discussion of epidemiological or health transitions in this paper, but briefly: there were major shifts in the proportionate mortality of NCDs and infectious diseases that align pretty well with major transitions in human behavior and culture in lots of places throughout the world, roughly aligning with the agricultural revolution, industrial revolution, and globalization. Before the industrial revolution in lots of places, NCD mortality was relatively small, although we don’t know that much about the prevalence. There is some big message in here for you to think about in your research and potentially acknowledge here, in which the ability to actually study NCDs via the oral microbiome to uncover the nature of NCDs in historical/pre-historic populations will be a major contribution to our understanding of health in those populations, since we have these preconceived notions about their relative prevalence using mortality as a proxy—which is probably not going to be as good of a proxy as actual biological data.
- Second paragraph on page 10: You discuss some limitations of using aDNA, specifically in that it is often fragmented and prone to contamination. Pathogens like *Mycobacterium tuberculosis, Mycobacterium bovis, Mycobacterium leprae, *etc. have been studied using molecular clock calibration with aDNA. Given the fact that you are discussing the bacteria that make up the microbiome throughout this article, would that method be useful for what you are describing here? Could it improve upon the fragmentation and contamination issues that plague other aDNA methods?

Is the review written in accessible language?

Yes

Are all factual statements correct and adequately supported by citations?

Yes

Are the conclusions drawn appropriate in the context of the current research literature?

Yes

Is the topic of the review discussed comprehensively in the context of the current literature?

Yes

Reviewer Expertise:

Infectious diseases, tuberculosis, influenza, co-morbidities, biocultural anthropology, syndemics, demographic transitions, epidemiological transitions

I confirm that I have read this submission and believe that I have an appropriate level of expertise to confirm that it is of an acceptable scientific standard, however I have significant reservations, as outlined above.

# Response

**Published:** 2023-04-04

**Authors:** Abigail Gancz

**Affiliations:** Pennsylvania State University, USA

**Reviewer 2**

 This paper reviews and synthesizes the literature linking the oral microbiome to various non-communicable diseases, and additionally provides some intriguing paths forward on how to study these connections in skeletal remains to illuminate another dimension of NCDs in past human populations. I really enjoyed this interesting, well-written, and well-researched paper, and I think the authors have significant contributions to make to the body of literature on ancient human health.

**We thank the reviewer for this summary of our research and their thoughts on its contribution to the field.**

 I have listed some of my more detailed thoughts below on the paper, some small and some more significant. Some of these comments are comments on mere spelling/style issues, but I have also included a couple larger items that I hope the authors consider, if not in this revision, then as the research progresses.

- First paragraph of introduction: Consider listing at least a couple of the seven of the top ten causes of death in 2019. Also, I do not suspect that the top ten causes of death would have changed substantially from 2019 to 2023 (other than the inclusion of COVID, of course), but do you have an updated source? I know that WHO has just a little bit of a lag, but there should be updated numbers from 2019 by now.

**We thank the reviewer for this astute comment. First, we have added examples (“Specifically, seven of the top ten causes of death in 2019 were attributed to NCDs (ex. heart diseases, diabetes, and cancers), and these accounted for 44% of all global deaths.”  We have also replaced the 2019 citation with the WHO 2022 report (although this still cites the same data from 2019, as that is the most up to date due to pandemic delays).**

  

- Second paragraph of introduction: One of the reasons listed for why the trend of studying NCDs through aDNA is that “infectious diseases can often be identified through distinctive mass-death burials or specific skeletal pathologies”. Certainly, for major epidemic/pandemic events, the mass death burials will be essential, but I do not agree that infectious diseases appearing in skeletal remains would be a major contributor to this. Overwhelmingly, acute infectious disease progression is too short to leave any real traces on skeletons. Infectious diseases like tuberculosis and leprosy are (sometimes) identifiable with some difficulty in skeletal material, but only after a very long period of infection and disease progression. Skeletal pathologies like markers of frailty could suggest potential susceptibility to acute infectious diseases, but infectious diseases overwhelmingly do not manifest in the skeleton. Because of the previous mention of Donoghue (2017), I suspect tuberculosis and leprosy are top of mind here, but this should be more carefully stated in the text.

**We thank the reviewer for these remarks. To address them, we have modified our phrasing to state “while individuals with severe cases of infectious diseases can sometimes be identified through distinctive mass-death burials or specific skeletal pathologies”. We completely agree that it is difficult to diagnose infectious diseases in ancestors using skeletal pathology unless individuals had long-lasting infections, and that this biases the observable record towards more severe cases. When speaking of the benefits of aDNA to infectious diseases, we are referring to the capacity of researchers to analyze genomes and traits associated with specific infectious agents, and we have now made that more clear via the following sentence: “Moreover, unlike with infectious diseases, aDNA cannot be directly used to establish the clear presence of a single pathogenic agent nor to study the genomes and traits associated with it”.**

  

- End of first paragraph of background: consider capitalizing “native” and “indigenous.”

**We thank the reviewer for this comment and have implemented the suggested changes.**

  

- Last full paragraph on page 4: The osteological paradox is mentioned very fleetingly, and although it is a popular concept and likely well known to your audience who studies skeletal material, it might be a good idea to define it briefly. Additionally, the citation of DeWitte & Stojanowski (2015) is good, but if you do dedicate space to the osteological paradox, it would be good practice to reference the original paper (Wood et al., 1992) as well.

**We thank the reviewer for this observation and have done the following: First, we have included the original paper (Wood et al 1992) in our citation of the osteological paradox. In addition, we have incorporated a definition: “the observation that deceased skeletal populations do not directly reflect the health or demographic characteristics of living populations”**.

  

- First paragraph on page 5 under “Cardiovascular disease”: in the list of conditions that make up CVD, “stroke” is listed twice.

**We thank the reviewer for this observation and have removed one instance of it.**

- First paragraph, 3 ^rd^ line on page 7: “ *Actinobacteira” *should be “ *Actinobacteria*”

**We thank the reviewer for catching this typo and have addressed it accordingly.**

  

- First paragraph in “Applying the present to the past” page 9: I see here that you do go ahead and cite the Wood et al. (1992) paper in reference to the osteological paradox! Just a small semantics thing in this sentence: I am not sure that the osteological paradox as a concept has “goals” *per se*, it would be more like the goals of understanding the nuances of the osteological paradox and the reality of the lived experiences of past populations.

**We thank the reviewer for this insightful comment and agree with the reviewer’s point. As such, we have modified our phrasing as follows: These advances are critical to pursue in order to advance approaches to the osteological paradox and improve paleoepidemiological research”**

  

- Second paragraph in “Applying the present to the past” on page 9: It occurred to me while reading this paragraph (which I really like, because it provides some clear steps forward), that the issue of the prevalence of NCDs in historical and/or prehistoric populations will be really dependent on how industrialized/urbanized/modernized a particular population is. I would not recommend going into a detailed discussion of epidemiological or health transitions in this paper, but briefly: there were major shifts in the proportionate mortality of NCDs and infectious diseases that align pretty well with major transitions in human behavior and culture in lots of places throughout the world, roughly aligning with the agricultural revolution, industrial revolution, and globalization. Before the industrial revolution in lots of places, NCD mortality was relatively small, although we don’t know that much about the prevalence. There is some big message in here for you to think about in your research and potentially acknowledge here, in which the ability to actually study NCDs via the oral microbiome to uncover the nature of NCDs in historical/pre-historic populations will be a major contribution to our understanding of health in those populations, since we have these preconceived notions about their relative prevalence using mortality as a proxy—which is probably not going to be as good of a proxy as actual biological data.

**We thank the reviewer for this comment and their insights. Indeed, a major reason we are pursuing this work is to enable a closer examination of health transitions across lifestyle shifts in different populations. Members of our team are currently working on the industrial revolution, agricultural revolution, and globalization in specific populations. To address your comment, we have modified the conclusion of our paper to touch upon these topics in more depth. **

- Second paragraph on page 10: You discuss some limitations of using aDNA, specifically in that it is often fragmented and prone to contamination. Pathogens like *Mycobacterium tuberculosis, Mycobacterium bovis, Mycobacterium leprae, *etc. have been studied using molecular clock calibration with aDNA. Given the fact that you are discussing the bacteria that make up the microbiome throughout this article, would that method be useful for what you are describing here? Could it improve upon the fragmentation and contamination issues that plague other aDNA methods?

**We thank the reviewer for this comment and have added the following clarification into the text: “While the genomes and evolutionary rates of well-studied microbes, such as those associated with pathogenic infections (ex. Mycobacterium tuberculosis, Mycobacterium leprae), are often better understood, much less information is available about understudied, commensal microbes such as those of the microbiome (Arriola et al. 2020).” To elaborate on this point, ancient microbiome studies differ from organism-specific studies in several ways, from the ways in which the DNA is captured to how it analyzed. For instance, while contamination is less of an issue for specific organism since researchers can (1) examine reads mapping directly only that organism and (2) authenticate it via DNA damage patterns against a reference, microbiome studies encompass both known and unknown organism. As such, contamination from modern sources is a far bigger issue. There are ways of addressing this (controls, decontam, source tracker, reference free authentication), and more are being developed. So, while ancient-pathogen studies share some of the same limitations as ancient microbiome studies, in some ways, microbiome studies can be more prone to these issues. However, much work is being done to improve methodologies related to these topics, including from researchers in our lab.**

# Peer review: Reviewer response for version 1

**DOI:** 10.5256/f1000research.141691.r161874

**Published:** 2023-02-14

**Authors:** Sarah Schrader

**Affiliations:** 1 Faculty of Archaeology, Leiden University, Leiden, The Netherlands

In this review contribution, Gancz and Weyrich present a compelling argument for how the oral microbiome might elucidate non-communicable diseases in the past. The authors draw on data from modern studies and couch this within potential applications to archaeological studies. More specifically, they address how cardiovascular disease, diabetes, rheumatoid arthritis, and Alzheimer’s disease interact with the microbiome and skeletal/dental lesions, such as periodontitis. The authors argue, and I agree, that this approach could significantly contribute to our understanding of frailty and the osteological paradox. A more thorough understanding of non-communicable diseases—a major culprit of morbidity and mortality around the world—through time is essential to tackling this problem. In the Challenges and Limitations sections, Gancz and Weyrich acknowledge that the diagnosis of specific non-communicable diseases in the past is unlikely; however, identifying microbiome markers associated with inflammation and non-communicable diseases is attainable. This is a very thought-provoking contribution that highlights the cutting edge of microbiome research and will certainly directly contribute to a better understanding of non-communicable disease through time and space.

Is the review written in accessible language?

Yes

Are all factual statements correct and adequately supported by citations?

Yes

Are the conclusions drawn appropriate in the context of the current research literature?

Yes

Is the topic of the review discussed comprehensively in the context of the current literature?

Yes

Reviewer Expertise:

Bioarchaeology; Archaeological Science; Paleopathology

I confirm that I have read this submission and believe that I have an appropriate level of expertise to confirm that it is of an acceptable scientific standard.

# Response

**Published:** 2023-04-04

**Authors:** Abigail Gancz

**Affiliations:** Pennsylvania State University, USA

We thank the reviewer for this thoughtful summary of our research.
